# Fragile X protein in newborn dried blood spots

**DOI:** 10.1186/s12881-014-0119-0

**Published:** 2014-10-28

**Authors:** Tatyana Adayev, Giuseppe LaFauci, Carl Dobkin, Michele Caggana, Veronica Wiley, Michael Field, Tiffany Wotton, Richard Kascsak, Sarah L Nolin, Anne Glicksman, Nicole Hosmer, W Ted Brown

**Affiliations:** Department of Developmental Biochemistry, New York State Institute for Basic Research in Developmental Disabilities, 1050 Forest Hill Road, Staten Island, New York, 10314 USA; Department of Human Genetics, New York State Institute for Basic Research in Developmental Disabilities, 1050 Forest Hill Road, Staten Island, New York, 10314 USA; Wadsworth Center, New York State Department of Health, Albany, New York USA; NSW Newborn Screening Programme & University of Sydney, Sydney, Australia; The NSW GOLD Service, Hunter Genetics, Newcastle, Australia

**Keywords:** Fragile X syndrome, FMR1, FMRP, Dried blood spots, DBS, Capture immune assay, CGG repeats, Luminex, Newborn screening

## Abstract

**Background:**

The fragile X syndrome (FXS) results from mutation of the FMR1 gene that prevents expression of its gene product, FMRP. We previously characterized 215 dried blood spots (DBS) representing different FMR1 genotypes and ages with a Luminex-based immunoassay (qFMRP). We found variable FMRP levels in the normal samples and identified affected males by the drastic reduction of FMRP.

**Methods:**

Here, to establish the variability of expression of FMRP in a larger random population we quantified FMRP in 2,000 anonymous fresh newborn DBS. We also evaluated the effect of long term storage on qFMRP by retrospectively assaying 74 aged newborn DBS that had been stored for 7-84 months that included normal and full mutation individuals. These analyses were performed on 3 mm DBS disks. To identify the alleles associated with the lowest FMRP levels in the fresh DBS, we analyzed the DNA in the samples that were more than two standard deviations below the mean.

**Results:**

Analysis of the fresh newborn DBS revealed a broad distribution of FMRP with a mean approximately 7-fold higher than that we previously reported for fresh DBS in normal adults and no samples whose FMRP level indicated FXS. DNA analysis of the lowest FMRP DBS showed that this was the low extreme of the normal range and included a female carrying a 165 CGG repeat premutation. In the retrospective study of aged newborn DBS, the FMRP mean of the normal samples was less than 30% of the mean of the fresh DBS. Despite the degraded signal from these aged DBS, qFMRP identified the FXS individuals.

**Conclusions:**

The assay showed that newborn DBS contain high levels of FMRP that will allow identification of males and potentially females, affected by FXS. The assay is also an effective screening tool for aged DBS stored for up to four years.

## Background

Fragile X syndrome, the most common inherited cause of intellectual disability, results from mutation of the *FMR1* gene on the X chromosome that disrupts expression of the fragile X mental retardation protein (FMRP) [1,2]. Although there are very rare cases in which the fragile X syndrome is due to point mutation or deletion of the *FMR1* gene [3–7], the most common fragile X mutation eliminates FMRP expression through expansion of a CGG repeat in the 5’-untranslated region of *FMR1* to more than 200 triplets (the full mutation). Although the fragile X syndrome results directly from the absence of functional FMRP, diagnosis of this syndrome is based on allele size and detection of the full mutation expansion in genomic DNA.

*FMR1* alleles are sorted into four size categories based on their stability upon transmission: normal (up to 44 CGG repeats): intermediate (45-54 repeats); premutation (55-200 repeats); and full mutation (>200 repeats). In the North American population the full-mutation allele (>200 repeats) which is exclusively maternally inherited, has an approximate prevalence of 1 in 4,000 while the premutation is much more common with a prevalence of 1 in 151 females and 1 in 468 males [8]. The premutation can expand to the full mutation when transmitted from mother to offspring and the likelihood of this expansion is dependent on the length and structure of the allele. The majority of premutation alleles are less than 90 triplet repeats in length. FMRP expression is reduced as triplet repeat length increases from the normal range and is eliminated by a process analogous to X inactivation when the repeat exceeds approximately 200 copies [9,10].

Since males carry only a single X chromosome, those with a full mutation allele develop the fragile X syndrome due to the absence of FMRP. Approximately 40% of males with the full mutation have some somatic cells with smaller alleles from triplet repeat contractions during early embryogenesis. These alleles express FMRP but only rarely does this mosaic expression ameliorate the syndrome. Contractions are also presumably responsible for the premutation size alleles in the sperm of full mutation males [11]. Large premutation alleles (approximately 150-200 repeats) are associated with intellectual impairment due presumably to reduced FMRP expression.

Females carry two X chromosomes and two copies of the *FMR1* gene, only one of which is expressed in any particular somatic cell after X inactivation during early embryogenesis. When FMRP expression is reduced because one *FMR1* allele in a female carries the full mutation, the degree of impairment can range from undetectable to severe. This variability is due to variation in random X inactivation and the resulting mosaic distribution of somatic cells in which FMRP is expressed. Since the full mutation must be maternally inherited, homozygous full mutation females do not occur. As in males, mosaicism for smaller alleles occurs in females but it is more rarely observed.

We recently reported the development of a simple, accurate, and inexpensive capture immunoassay that determines the level of FMRP in dried blood spots (DBS) as well as in lymphocytes and other tissues [12]. Our initial study of FMRP in DBS from 215 individuals with normal, premutation, and full-mutation *FMR1* alleles was designed to characterize the FMRP expression of different *FMR1* genotypes. In samples from normal individuals we found a broad distribution of FMRP. The level of the protein declined with age from infants to preteens. It leveled off in teenage years and remained unchanged through adulthood, with no difference between males and females. We used these DBS samples to evaluate how effectively this assay distinguished affected from unaffected individuals. While the assay readily identified affected (full-mutation) males with sensitivity and specificity approaching 100%, we needed to test a larger set of random population samples to establish the FMRP variability detected by the assay. Since residual DBS from state-mandated newborn screening for metabolic and genetic diseases are available for research and represent a uniform age, we decided to use 2,000 randomly selected anonymous fresh newborn DBS to characterize the variability of FMRP expression in the newborn population which is a potential target for screening with this assay. Considering the estimated prevalence of fragile X, it was relatively unlikely that we would find any affected individuals (i.e. those with virtually no FMRP) among the 1,000 male and 1,000 females sampled. We were primarily interested, however, in the variability of FMRP expression, especially the low extreme of normal expression.

To evaluate the effect of long term storage of newborn DBS on the qFMRP assay, we conducted a retrospective study of 74 aged newborn DBS that had been stored for up to 7 years. These DBS were from a different newborn screening program and included samples from 6 affected (full mutation) males.

## Methods

Newborn DBS disks (3-mm-diameter; ~7.1 mm^2^) were obtained from the New York State Department of Health’s Wadsworth Center, Albany, NY, USA and the New South Wales Newborn Screening Program, Sydney, Australia. Studies performed with both sets of disks were reviewed and approved by the Institutional Review Boards of the Institute for Basic Research in Developmental Disabilities (IBR) and the DBS source institutions.

### Newborn DBS from the Wadsworth center in New York State

The DBS disks from 1,000 males and 1,000 females were recent (5 weeks old) and stored with a desiccant in a refrigerator at 2-8°C. They were received (in duplicate) in sealed 96-well plates with no identifying information except gender.

### Newborn DBS from New South Wales (Australia) newborn screening program

The seventy-four disks from DBS that had been stored for 7 to 84 months included samples from 6 males diagnosed with the fragile X syndrome and 68 normal individuals. The samples from 6 affected males had been stored from 19 to 79 months at low humidity and 22-28°C for 12 months and then at 20°C before FMRP quantification. Those from 68 normal controls had been stored from 7 to 84 months. Information about phenotype, gender, and age of DBS was not known by the researchers performing qFMRP analysis until completion of the study.

### Elution of FMRP from DBS

Each 3-mm-diameter disk was placed into a well of a Low Protein Binding Durapore R Multiscreen 96-well filter plate (Millipore, Billerica, MA) and protein was eluted in 50 uL of extraction reagent: M-PER mammalian protein extraction reagent (Thermo Fisher Scientific, Rockford, IL) containing 150 mmol/L NaCl, 10 ug/mL chymostatin, 10 ug/mL antipain, and 1× protease inhibitor cocktail (Complete mini tablets, EDTA free; Roche Applied Science, Indianapolis, IN) by shaking for 3 hr with agitation at room temperature. Eluates were collected by centrifugation at 4°C into a 96-well catch plate for 5 minutes at 1258 × g [12].

### qFMRP assay procedure

FMRP assays were performed with the anti-FMRP mouse monoclonal antibody mAb6B8 (MMS-5231, Covance Inc., Dedham, MA) and the anti-FMRP rabbit polyclonal antibody R477 [12]. These antibodies are highly specific and each recognizes a different epitope of the protein [12]. A GST fusion protein, GST-SR7 carrying an abbreviated sequence of FMRP that includes the epitopes of mAb6B8 and R477 was used as standard [12]. The immunoassays were performed as previously described [12]. Briefly, 50 uL DBS eluate was incubated for 6 hr with 3,000 xMAP-MicroPlex microspheres (Luminex, Austin, TX) coupled to anti-FMRP mAb6B8. The microspheres were then washed and incubated overnight with rabbit antibody, R477 that was subsequently labeled for 2 hr by phycoerythrin-conjugated goat anti-rabbit IgG. FMRP was quantified with a Luminex 200 system. Dilutions of GST-SR7 were used to generate an FMRP standard concentration curve for each 96-well plate analyzed. The amount of FMRP in the DBS was reported as concentration (pM) in the 50 uL DBS eluate.

### DNA studies

DNA was eluted from approximately ½ of a duplicate 3mm (~3.5 mm^2^) disk by the following modification of a published procedure [13].The DBS disk was initially washed in 1 mL of SSPE (0.15 M NaCl, 0.01M NaH_2_PO_4_ pH 7.0, and 0.001M EDTA) containing 0.1% Tween 80 (Sigma-Aldrich, St Louis, MO 63103 USA) for 10 minutes at room temperature and the half disk was transferred to 100 μL of 5% Chelex (BioRad, Hercules, CA 94547 USA) in H_2_O, incubated for 30 min at 60°C and then for 30 min at 100°C. The liquid phase was separated from the Chelex and brought to 1 mM EDTA. Two microliters of this eluate served as template for polymerase chain reaction (PCR) amplification of the *FMR1* CGG repeat region with the AmplideX® *FMR1* PCR (RUO) reagents according to the manufacturer’s directions (Asuragen, Austin, TX 78744 USA). PCR products were analyzed by capillary electrophoresis (ABI 3130 Genetic Analyzer, Applied Biosystems, Foster City, CA) [14]. This eluate was also used for analysis of DNA methylation in the *FMR1* CGG repeat region with the Amplidex® *FMR1* mPCR kit according to the manufacturer’s directions (Asuragen, Austin, TX 78744 USA).

### Statistical analysis

Data were analyzed with either IBM SPSS Statistics (IBM, Armonk, NY) or SigmaPlot (Systat Software, San Jose, CA) software.

## Results

### NY State newborn DBS samples

**Figure 1:**
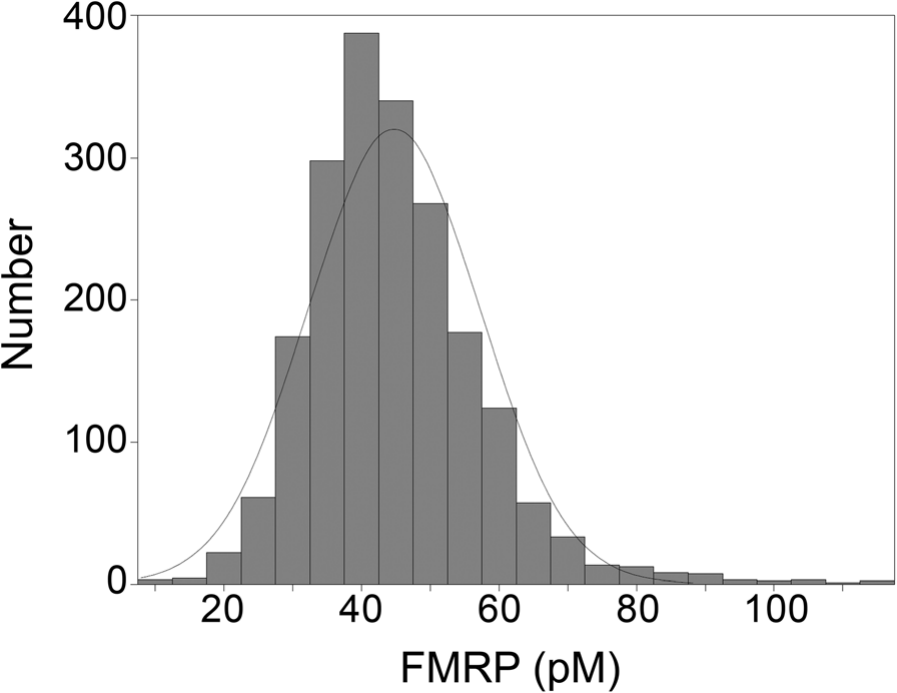
**Distribution of FMRP levels in 2,000 newborn DBS from the NY State collection.** The mean FMRP value was 44.8 pM; standard deviation 12.4 pM; skewness 1.11; kurtosis 3.175.

We applied the qFMRP immunoassay to 1,000 male and 1,000 female newborn DBS that had been stored for only five weeks (fresh DBS). FMRP concentration (pM) in each sample (3-mm-diameter disk eluate) was calculated by comparison to dilutions of GST-SR7 as previously described [12]. The results showed a variable expression of FMRP ranging from 10.3- to 92-pM, an average FMRP of 44.8 pM, and a SD of 12.4 pM (Figure 1). Comparison of the FMRP levels obtained in this study to those reported previously [12] for normal adults using larger DBS disks (6.9-mm-diameter, 37.4 mm^2^) revealed that, the average FMRP in newborn was approximately seven-fold higher (6.3 pM eluted per mm^2^) than in adults (0.93 pM eluted per mm^2^). None of the 1,000 male or 1,000 female random newborn DBS lacked FMRP or had the extremely low level that would indicate the fragile X syndrome [12].

**Figure 2:**
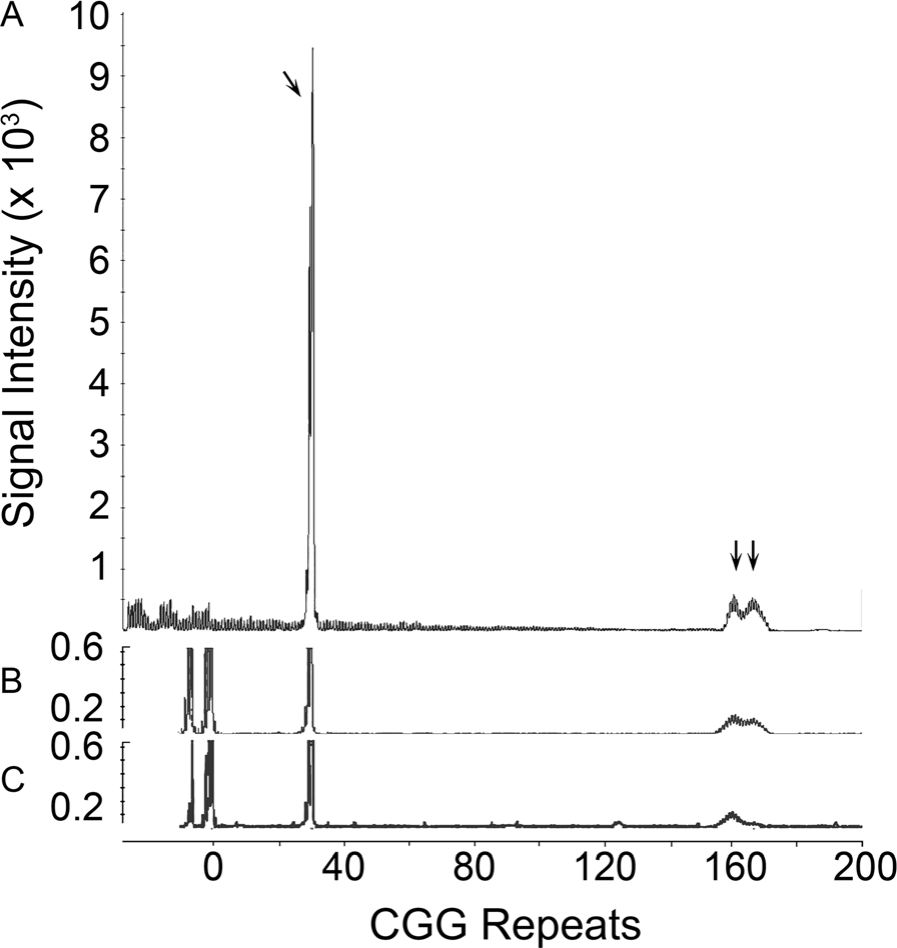
**PCR analysis of DBS sample 2 in Table** **1** **, a female with a large premutation allele and a highly methylated normal allele. A**: Capillary electrophoresis profile of PCR analysis. Arrows indicate alleles of 30, 161 and 167 CGG repeats. The 161 and 167 repeat alleles (arrows at right) represent a premutation allele. (Somatic mosaicism is presumably responsible for the bifurcation). **B**: Methylation analysis reference (no Hpa II digestion) PCR profile of DBS DNA. **C**: Methylation analysis PCR profile of Hpa II-digested DBS DNA. Comparison of **B** and **C** illustrates how each allele is protected from HpaII digestion by methylation. The premutation was split into two alleles and the analysis suggests that the larger, 167 repeat had a lower level of methylation than the smaller, 161 repeat allele. The 2 PCR products at approximately 0 CGG repeats represent internal controls for the methylation analysis.

To examine the *FMR1* genotypes of the samples at the low extreme of the FMRP distribution, we extracted genomic DNA from duplicate DBS of the 14 samples whose level of FMRP was more than two standard deviation units below the newborn population mean and determined the size of the CGG repeat in the *FMR1* alleles that were present (Table 1). The alleles detected in the 14 DBS samples (0.7%) that met this criterion are shown in Table 1. Thirteen of the samples showed an assortment of normal *FMR1* CGG repeat alleles that reflects the allele distribution in the normal population which has a mode of 30 repeats. One sample from a female at the extreme low end of the FMRP distribution (Table 1, Sample 2) showed a normal allele of 30 repeats and a large premutation that appeared as two alleles of approximately 161 and 167 repeats (Figure 2). Methylation analysis, informed by two HpaII sites on either side of the CGG repeat [15,16] showed that the normal, 30 repeat, allele was highly resistant to Hpa II digestion which indicated that it was highly (~90%) methylated (Figure 2). This implied highly skewed X inactivation in which the normal allele resided on the inactive X chromosome in most white blood cells in the newborn blood sample while the premutation resided on the active chromosome in most of these cells.

**Table 1:** **NY State newborn DBS with lowest FMRP levels**

**Sample**	**FMRP(pM)**	**Z** ^**#**^	**Sex**	**Allele 1**	**Allele 2**	**Allele 3**
1	10.3	−2.8	f	32	44	
2	10.4	−2.8	f	30	161	167
3	11.3	−2.7	m	20		
4	13.2	−2.5	m	31		
5	16.6	−2.3	m	30		
6	16.9	−2.2	m	30		
7	17.5	−2.2	m	29		
8	17.8	−2.2	f	22	23	
9	18.6	−2.1	m	29		
10	19.0	−2.1	f	30	33	
11	19.2	−2.1	m	29		
12	19.2	−2.1	m	30		
13	19.6	−2.0	m	30		
14	19.7	−2.0	m	30		
Mean*	44.8					
SD*	12.4					

*Mean and standard deviation of all 2,000 samples.

#Difference from mean (in standard deviation units).

Allele sizes are in CGG repeat number.

### New South Wales newborn DBS samples

**Figure 3:**
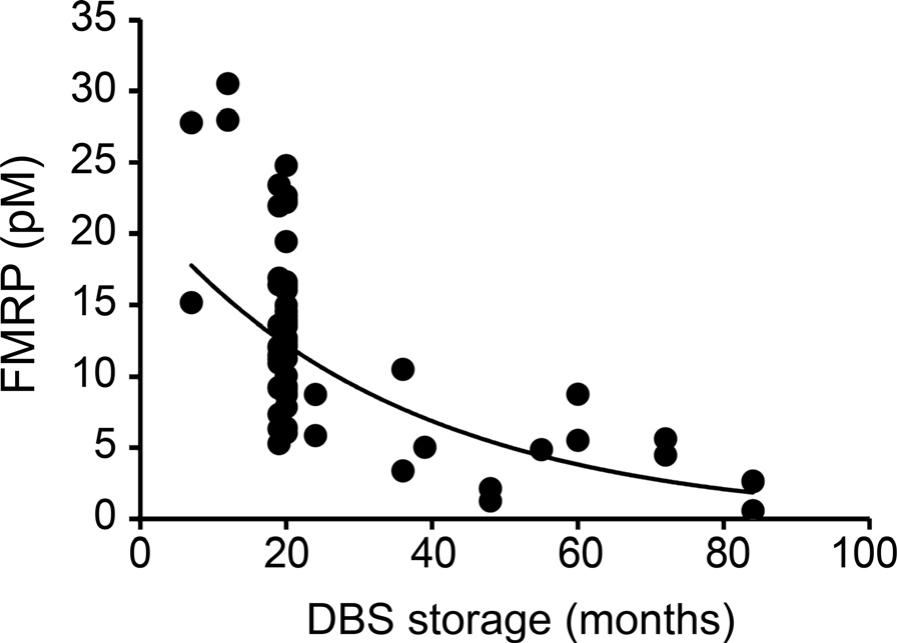
**Decline in detectable FMRP with DBS storage time.** Samples from normal individuals are plotted according to duration of storage in months. The formula for the best fit trend line: y =21.903e^-0.028×^; R^2^ = 0.5509.

**Figure 4:**
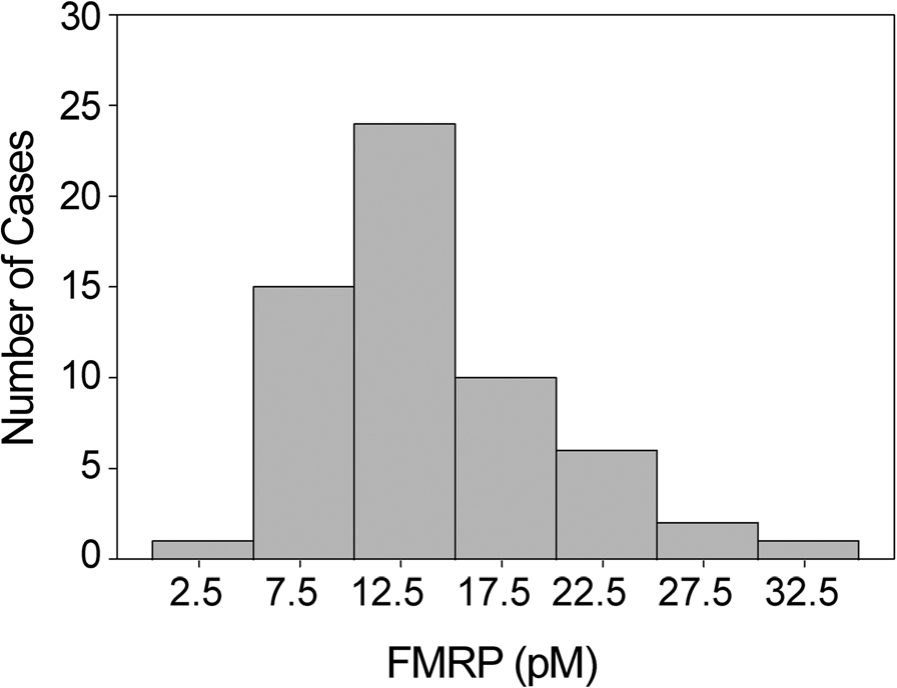
**Distribution of FMRP levels in 59 newborn DBS from normal controls in the New South Wales archive.** The mean FMRP value was 12.5 ± 6.5. Storage time for this sample set was ≤47 months.

**Figure 5:**
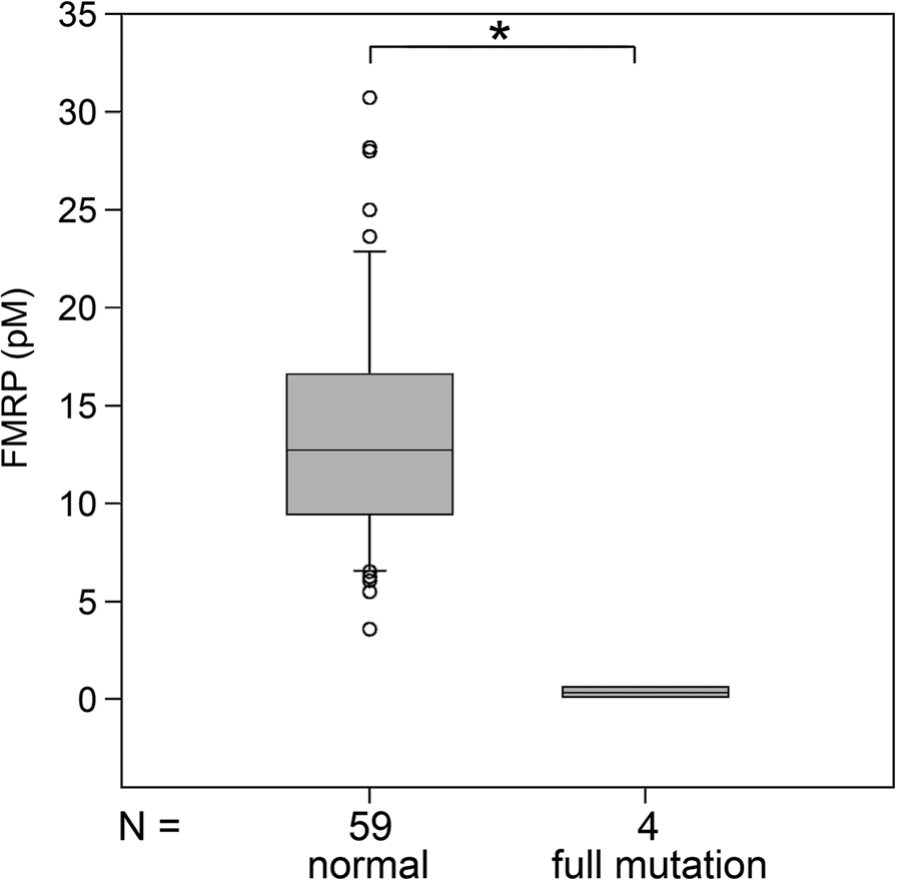
**Boxplot of FMRP values in 63 newborn DBS from New South Wales archive.** Box-plot data are expressed as 25th to 75th percentile, median, and whiskers to 10^th^ and 90^th^ percentiles with outliers shown as circles. *P = ≤0.001, Mann-Whitney U-test. Storage time for this sample set was ≤47 months.

We also applied the qFMRP assay in a retrospective study of 74 newborn DBS that had been stored for an extended period and included 6 full mutation males as well as 68 normal individuals. This analysis was performed in a blinded manner to correlate the FMRP levels detected in the aged newborn DBS with the diagnoses of the fragile X syndrome that had been made later by the GOLD Service Hunter Genetics (Newcastle, Australia), after the phenotype appeared. The storage time for the full mutation and normal DBS ranged from 19 to 79 months and from 7 to 84 months, respectively. Table 2 shows the results for the 9 aged newborn DBS with the lowest levels of FMRP. The FMRP level of the 6 full mutation males in this sample set was indistinguishable from background and more than 20 fold lower than the average normal level in this aged sample set. Analysis of the normal controls in this study indicated that the amount of detectable FMRP in the DBS had declined significantly with extended storage (Figure 3) which shifted the FMRP distribution toward zero (Figure 4). The only normal sample that approached (0.77 pM) the level of the fragile X syndrome samples had been stored for seven years. Despite the loss of detectable FMRP with DBS storage time, the qFMRP assay identified all of the affected (full mutation) males. Because of the decline of detectable FMRP in normal control DBS that could lead to false positives, the Mann-Whitney analysis and the boxplot of these results shown in Figure 5 excluded samples that had been stored for more than 47 months. Samples in this subset showed a significant separation between normal (male and female) and full mutation males (P ≤0.001, U-test).

**Table 2:** **Australian newborn DBS with lowest FMRP levels**

**Genotype**	**Sex**	**FMRP pM**	**zFMRP** ^**#**^	**Storage (mo)**
full	m	0.11	−1.9	47
full	m	0.14	−1.9	19
full	m	0.35	−1.9	73
full	m	0.53	−1.8	38
full	m	0.58	−1.8	79
full	m	0.64	−1.8	32
nl	f	0.77	−1.8	84
nl	m	1.45	−1.7	48
nl	f	2.32	−1.6	48
Mean*	m + f	12.5		
SD*		6.5		

*Mean and standard deviation of control normal samples (n = 59).

#Difference from mean (in standard deviation units).

nl: normal, full: full mutation.

## Discussion

The distribution of FMRP levels in the sample of 2,000 newborn DBS from the NY State collection (Figure 1) parallels the profile of 134 non-newborn DBS samples from individuals of normal phenotype and normal size CGG repeat alleles that were analyzed previously [12]. The mean FMRP level was considerably higher than expected--approximately seven-fold higher than in the previous study of 134 normal individuals. While the higher white blood cell level in newborns [17] is almost certainly a contributor to this increase, only about half of the increase in the average FMRP level is explained by the predicted increase in white blood cell count. This suggests that newborns may have increased FMRP expression in white blood cells. Whatever the explanation, the increase in the average FMRP level will enhance the specificity and sensitivity of the qFMRP assay for screening newborn DBS.

Considering the prevalence of fragile X, it is not surprising that none of the 1,000 samples from males and 1,000 from females lacked FMRP or had an FMRP level low enough to signal the presence of the fragile X syndrome. Although there were clearly no samples in this set of 2,000 that were from individuals who would develop the fragile X syndrome, we examined the *FMR1* CGG region in the DBS samples with FMRP lower than two SDs below the mean to see what alleles were present at the low extreme of the distribution. Thirteen of the 14 samples in this group had normal CGG repeat alleles, which confirmed that these FMRP levels were within the normal range and that they contained reduced levels of FMRP due to factors other than CGG repeat length—for example, due to the variability in the number of white blood cells in newborns [18].

In contrast one sample (sample 2 in Table 1) appeared to be at the low extreme of the normal FMRP distribution due to reduced FMRP expression from a 161/167-repeat premutation allele. *FMR1* alleles of this size are rare and have reduced levels of FMRP [9,19]. Methylation analysis (Figure 2) indicated skewed inactivation of approximately 90% of the X chromosome with the normal 30-repeat allele. Thus, FMRP expression was primarily from the premutation allele with its reduced FMRP expression which is probably the explanation for the low-normal amount of FMRP. The assignment of this one sample out of 2,000 to the low normal range is extremely unlikely to have occurred by chance and demonstrates the potential discriminatory power of this assay.

The aged DBS from New South Wales newborn screening collection were very different from the fresh 2,000 DBS from the NY State collection. The former newborn DBS had been archived for an extended period and were analyzed for FMRP only after some of the males represented were later found to have the fragile X syndrome. The extended storage time reduced the level of FMRP that could be detected (Figure 3) which shifted the distribution of FMRP levels toward zero (Figure 4). The mean normal concentration was approximately half that of the normal adult population we had previously analyzed [12] and less than a third of the mean of newborn DBS that had been stored for only five weeks (Table 1). The results shown in Table 2 indicate that extended DBS storage, especially for longer than 48 months, could lead to some overlap between normal and full mutation. Despite the reduction of detectable FMRP due to prolonged storage, this retrospective analysis was a highly effective screen that identified all specimens from affected (full mutation) males. However, prolonged storage of newborn DBS (more than 47 months) could lead to an increase in false positive samples. Thus, the qFMRP assay will have limited utility for DBS stored for four years or more. Future retrospective studies with aged newborn DBS should be performed with samples that have been stored less than 47 months, and the levels of FMRP should be compared to those of aged normal DBS having the same prolonged storage time.

It is likely that the qFMRP assay would be able to predict cognitive impairment in full mutation females and distinguish between those that had an IQ <70 and high functioning full mutation as well as premutation females. Studies of *FMR1* alleles have shown that the methylation of specific sites is predictive of intellectual impairment in full mutation females [20,21]. This methylation is presumably an indirect measure of FMRP expression and thus a direct qFMRP analysis is likely to be as predictive as an assay of *FMR1* methylation.

Fragile X does not have a distinctive phenotype in infants and young children and the average age at which the syndrome is diagnosed in males is 36 months in the USA and 54 months in Australia [22–24]. While there is currently no specific treatment for fragile X, early diagnosis of the syndrome would allow early therapeutic intervention for affected children and timely genetic counseling for their parents. Early diagnosis would become even more critical if pharmacological therapies for fragile X that are currently in phase 2 or 3 clinical trials prove effective.

Diagnosis of the fragile X syndrome is currently based on analysis of the CGG repeat in genomic DNA and DNA molecular tests have been used in pilot newborn screening for fragile X [25,26]. However, DNA molecular tests that determine CGG allele size identify premutation carriers as well as full mutation individuals. Premutation carriers are at risk for an adult onset disorder, fragile X associated tremor/ataxia syndrome (FXTAS) [27]. Since there is currently no treatment for FXTAS, the identification of newborns with the premutation complicates the ethics of fragile X screening by DNA analysis. There are currently no fragile X newborn screening programs.

## Conclusion

Our data show for the first time that it is feasible to measure FMRP in 3-mm-diameter newborn DBS disks which are used in mandatory screening for metabolic and hereditary diseases. The accurate measurement of FMRP in these small diameter disks is enhanced by the relatively high levels of the protein present in neonates, which could in part be due the high leukocyte count [17,18].

The level of FMRP in newborn is variable and is, on average, seven times higher than that detected in adults. Even though the levels of FMRP detected in DBS decrease with storage time, the qFMRP assay allowed us to identify all affected males from the set of aged DBS from normal and fragile X individuals. Our data suggests that the qFMRP assay could serve as the initial step in a fragile X newborn screening program. In a second screening step, characterization of CGG size and/or methylation status of the FMR1 alleles associated with DBS at the low extreme of the FMRP distribution would indicate the presence of the fragile X syndrome. The correlation between highly reduced or absent FMRP expression and the fragile X syndrome is firmly established for males. This correlation is likely to apply to females as well but further studies will be necessary to firmly establish this link. Fragile X screening by qFMRP has distinct advantages over techniques that detect a CGG expansion since it avoids ethical issues associated with identification of asymptomatic premutation carrier infants and is consistent with current newborn screening technology and cost parameters.
